# Importance of revising a psychiatric diagnosis over time in children with neurodevelopmental disorders: a case report of a girl with Asperger’s syndrome and intellectual disability

**DOI:** 10.1192/j.eurpsy.2025.1128

**Published:** 2025-08-26

**Authors:** M. Fazlovic, N. Zigic

**Affiliations:** 1 Department of Psychiatry, Public Health Institution “Health Center Brčko” Hospital, Brčko; 2 Department of Psychiatry, University Clinical Center, Tuzla, Bosnia and Herzegovina

## Abstract

**Introduction:**

Autistic spectrum disorders belong to the group of neurodevelopmental disorders, where the manifestations of the disorder differ depending on the severity of the autistic condition. These disorders are often associated with intellectual impairments and structural language disorders. In his patients, Asperger described cases with above-average intelligence as well as cases with low language and intellectual abilities. What they had in common was that they had significant disorders in social and affective communication. The diagnosis of autism spectrum disorders is usually made in childhood, but there are also cases where the diagnosis is made at an older age due to the later manifestation of symptoms.

**Objectives:**

To present a case report of 18 years old girl who was diagnosed with moderate intellectual disability in childhood, while later established with symptoms of Asperger’s syndrome and mild intellectual disability.

**Methods:**

Psychiatric interview, medical history, psychological testing

**Results:**

An 18-year-old girl, accompanied by her mother, presented herself to psychiatrist for the first time due to affective disorders that manifested in the form of increased nervousness and outbursts of anger. Early psychomotor development was slow, she started walking at the age of 1.5 years and spoke at the age of two. From early childhood, stereotypies in movements and hypervigilant attention were observed, along with very poor social interactions. Due to difficulties at school, she was categorized at the age of 12 as having moderate intellectual insufficiency and IQ of 35. She underwent long-term speech therapy treatment and finished elementary and high school with the help of teaching assistant. Somewhere in high school, the patient’s pronounced talent for drawing was noticed. Observation during the psychiatric examination reveals emotional immaturity, stereotypes and rigidity in social interactions. The patient is referred again for psychological testing, results show IQ of 88 with a lag in emotional development and development of basic social skills. The patient is referred to another psychiatrist for an additional opinion, who agrees with diagnose of Asperger’s syndrome. Low dose of lamotrigine was included in therapy, after which affective state stabilized. The patient is referred again for categorization.

**Conclusions:**

This case report that a diagnosis established in childhood period does not have to be definitive and that revision of diagnosis is necessary over time and as necessary, initially due to increase of patient’s IQ, as well as due to the later presentation of symptoms characteristic of certain disorder.

**Disclosure of Interest:**

None Declared

